# Supplementary material for: Comparison of three dosing intervals for the primary vaccination of the SARS-CoV-2 mRNA Vaccine (BNT162b2) on magnitude, neutralization capacity and durability of the humoral immune response in health care workers: A prospective cohort study
Source: PLoS One. 2023 Feb 15;18(2):e0281673. doi: 10.1371/journal.pone.0281673 (PMC9931154; doi:10.1371/journal.pone.0281673)
Supplement: S1 File — (DOCX) [file pone.0281673.s001.docx]

**SUPPLEMENTAL MATERIALS.**

**Comparison of three dosing intervals for the primary vaccination series of SARS-CoV-2 mRNA Vaccine (BNT162b2)** **on magnitude, neutralization capacity and durability of the humoral immune response in health care workers: a prospective cohort study.**

**S1 APPENDIX.**

**IMMUNE ASSAYS AND THRESHOLD OF DETECTION**

**Enzyme-linked immunosorbent assay (ELISA) for the measurement of serum anti-Spike, and anti-RBD SARS-CoV-2 antibodies.**

Plasmids encoding mammalian cell codon optimized sequences for SARS-CoV-2 full-length Spike protein and Receptor Binding Domain (RBD) were produced as described previously^1^. These proteins were used in a SARS-CoV-2 enzyme-linked immunosorbent assay (ELISA) measuring COVID-19 antibodies (IgG, IgA and IgM for Spike/RBD) as described previously^2^. Briefly, microtiter well plates (384 wells, Nunc Maxisorp) were coated overnight at 4ºC with 25 µL/well of Spike protein (5 µg/mL), and RBD (2 µg/mL), in 50 mM carbonate-bicarbonate buffer (pH 9.6). Plates were then blocked for 2 hours at room temperature with 3% skim milk in phosphate buffered saline (PBS) with 0.05% Tween 20. Plates were washed twice with PBS and 0.05% Tween 20, followed by three further washes with PBS alone. After washing, serum samples diluted (1/100) in 1% skim milk in PBS with 0.05% Tween 20 were added to duplicate wells and incubated for 1 hour at room temperature. Next, the plates were washed as above. Bound human IgG, IgA and/or IgM were detected with alkaline phosphatase conjugated goat anti-human 1/2000 IgG, 1/500 IgA, and 1/1000 IgM (Jackson ImmunoResearch Laboratories Inc) prepared in 1% skim milk in PBS with 0.05% Tween 20. Plates were washed, followed by the addition of substrate, 4-nitrophenylphosphate disodium salt hexahydrate in diethanolamine (MilliporeSigma). Optical density (OD) was read at 405 nm using a BioTek 800TS microplate reader (BioTek). The assay thresholds used to determine seropositivity have been previously determined by our laboratory as the mean plus 3 standard deviations of a pre-COVID-19 control population from the same geographic region^2^.

**Assay threshold of detection:**

| **Antibody isotypes** | **Anti-Spike** | **Anti-RBD** |
| --- | --- | --- |
| IgG | 0.549 | 0.551 |
| IgA | 0.544 | 0.564 |
| IgM | 0.582 | 0.598 |

**Microneutralization Titre at 50% (MNT50) Assay for the measurement of serum antibody neutralization of live SARS-CoV-2** **ancestral and Beta (B.1.351) variants.**

VeroE6 cells (ATCC CRL-1586) were seeded onto white flat-bottom 96-well plates (Costar) at a density of 2.5x10^4^ cells per well for 24 hours in complete DMEM (supplemented with 10% FBS, 1% L-glutamine, 100U/mL penicillin-streptomycin). Participants’ sera were inactivated at 56ºC, and serially diluted in a 1:2 dilution series starting at a 1:10 dilution, in low serum DMEM (supplemented with 2% FBS, 1% L-glutamine, 100 U/ml penicillin-streptomycin), to a final volume of 55 µL, into 96 well U-bottom plates. Live viruses of ancestral (SARS-CoV-2/SB3-TYAGNC)^3^ or B.1.351 variant (BEI Resources, NIAID, NIH: SARS-Related Coronavirus 2, Isolate hCoV-19/South Africa/KRISP-K005325/2020, NR-54009, contributed by Alex Sigal and Tulio de Oliveira) were diluted in low DMEM, to a final concentration of 6000 plaque forming units (PFU)/mL. Diluted virus was added to diluted serum at a 1:1 ratio. The viral-serum mixture (containing 330 PFU per well) was incubated at 37ºC for 1 hour and then 100 µL was added to the VeroE6 cultured cells. After 72 hours incubation at 37ºC, 50 µl of culture supernatant was replaced with 50 µl of CellTiter-Glo 2.0 Reagent (Promega, G9243). Plates were shaken at 282 cpm at 3mm diameter for 2 min, and incubated for 5 min, before luminescence was read using a BioTek Synergy H1 microplate reader with gain of 135 and integration time of 1 second. Data are reported as geometric microneutralization titers at 50% (MNT50), which ranged from below detection (MNT50 = 5, 1:10 dilution) to MNT50 = 1280

**Detection of SARS-CoV-2 RBD-specific memory B cells by ELISPOT**

Heparinized peripheral blood was spun at 514 x g for 10 minutes to separate plasma. Plasma depleted blood was then diluted with equal volume of PBS, layered over Histopaque-1077 (Millipore-Sigma, St Louis, MO, USA) and separated by a density gradient centrifugation at 1160 x g for 10 minutes. Once the buffy coat was removed, the sample was washed with PBS then spun at 514 x g for 10 minutes. The peripheral blood mononuclear cells (PBMC) pellet was resuspended in PBS and quantified using a Bio-Rad TC20 automated cell counter with trypan blue dye exclusion (Bio-Rad, Hercules, CA, USA). Next, PBMC were spun down at 514 x g for 10 mins and resuspended in 90% Human AB male serum (Millipore-Sigma) and 10% DMSO (Millipore-Sigma) for storage.

For experiments, PBMC (10 million cells) were spun at 350 x g for 10 minutes and resuspended in 5 mL of pre-warmed RPMI with 10% FBS, 2 mM L-glutamine, 100 units/mL penicillin, 100 μg/mL streptomycin and 1 U/mL heparin. The PBMC were quantified using a Bio-Rad TC20 automated cell counter with trypan blue dye exclusion, spun again at 350 x g for 10 minutes and resuspended to a final concentration of 3x10^6^/mL using serum free AIM-V media (Life Technologies-Gibco) with 2 mM L-glutamine, 100 units/mL penicillin and 100 μg/mL streptomycin. An equal volume (500 µL) of thawed PBMC resuspended at 3x10^6^/mL in AIM-V (with 2 mM L-glutamine, 100 units/mL penicillin and 100 μg/mL streptomycin) were stimulated with an equal volume (500 µL) of 4 µg/mL (2 µg/mL final) R848 (Resiquimod; Mabtech, Stockholm, Sweden) and 20 ng/mL (10 ng/mL final) IL-2 (Mabtech) or unstimulated PBMC were cultured in (500 µL) AIM-V media alone in 24-well tissue culture plates (Corning, New York, NY, USA) then incubated at 37°C, 5% CO_2_ for 5 days. After 5 days, stimulated and unstimulated PBMC were collected and spun at 350 x g for 10 minutes. Supernatants containing secreted antibodies were collected and frozen at -80ºC. The pelleted PBMCs were then resuspended and washed three times with AIM-V serum free media with 2 mM L-glutamine, 100 units/mL penicillin and 100 μg/mL streptomycin. Finally, stimulated and unstimulated PBMCs were once again quantified using a Bio-Rad TC20 automated cell counter with trypan blue dye exclusion. SARS-CoV-2 RBD-specific memory B cells were detected using a commercially available ELISPOT kit (Mabtech) following manufacturer’s instructions. Briefly, ethanol-treated polyvinylidene fluoride (PVDF) membrane ELISPOT plates coated with anti-IgG were washed with PBS (200 μL/well) then conditioned with RPMI supplemented with 10% FBS (200 μL/well) and incubated for 1 hour at room temperature. After removing the media, unstimulated and stimulated PBMCs were then added to the conditioned plate at 200,000 cells/well in duplicate whereas 10,000 cells/well of stimulated cells were added in duplicate to assess total IgG secretion and incubated at 37°C, 5% CO_2_ overnight. The following day, cells were removed and the plates were washed five times with PBS (200 μL/well). Secreted IgG was detected by either recombinant RBD-Wiskott Aldrich Syndrome Protein (WASP; 100 μL/well) for SARS-CoV-2 specific wells or biotinylated anti-IgG (100 μL/well) for total IgG wells and incubated for 2 hours at room temperature. Next, plates were washed five times with PBS (200 μL/well) then RBD-specific IgG wells were reacted with anti-WASP-horseradish peroxidase (HRP;100 μL/well) and total IgG wells were reacted with streptavidin-HRP (100 μL/well) and incubated for 1 hour at room temperature. The plates were once again washed five times with PBS, then 3,3',5,5'-Tetramethylbenzidine (TMB) substrate (100 μL/well) was added and allowed to develop until distinct spots emerged. Finally, colour development was stopped by washing with deionized water and plates were allowed to dry. ELISPOT wells were automatically counted using a CTL Immunospot Analyzer (CTL, Shaker Heights, OH, USA) and frequencies of specific memory B cell were calculated from the ratio of cells secreting SARS-CoV-2 RBD specific IgG antibodies versus all IgG-secreting cells.

**References:**

1. Stadlbauer D, Amanat F, Chromikova V, et al. SARS-CoV-2 Seroconversion in Humans: A Detailed Protocol for a Serological Assay, Antigen Production, and Test Setup. *Curr Protoc Microbiol*. 2020;57(1):e100
2. Huynh A, Arnold DM, Smith JW, et al. Characteristics of anti-SARS-CoV-2 antibodies in recovered COVID-19 subjects. *Viruses* 2021;13:697.
3. Baerjee A, Nasir JA, Budylowski P et al. Isolation, sequence, infectivity, and replication kinetics of Severe Acute Respiratory Syndrome Coronavirus 2. *Emerg Infect Dis* 2020;16:2054-2063.

**S2 APPENDIX.**

**Number of self-reported confirmed COVID-19 infections with data censored from the analysis.**

313 HCW enrolled

**number excluded due to COVID-19**

**Second dose visit**

>42 days schedule

n=2

<35 days schedule

n=0

35-42 days schedule

n=1

**3 week visit**

**number excluded due to COVID-19**

>42 days schedule

n=0

35-42 days schedule

n=1

1

<35 days schedule

n=1

**number excluded due to COVID-19**

**3 month visit**

>42 days schedule

n=2

35-42 days schedule

n=3

<35 days schedule

n=0

0

**number excluded due to COVID-19**

**6-9 month visit**

<35 days schedule

n=2

35-42 days schedule

n=1

>42 days schedule

n=7

7

**S3 APPENDIX:**

**Sample size calculation.**

The study began with 2 primary objectives which were to examine for (1) differences in anti-Spike IgG at <35-days, and 35-42 days following the first dose and prior to the second dose administration; and (2) detecting for significance of the groups*visits interaction term to inform differences in binding antibody kinetics by dosing intervals.

For the first objective, the hypothesis was that there will be an inverse association between the number of days between the first and second doses of vaccine and the immune response as measured by anti-Spike IgG at the time of the second dose administration. The assumptions made were: that the ln(geometric mean titer) among those with a shorter (<35-day) between-dose interval was 12.61 (95% CI 12.24 -12.99) with SD 0.74 and that the geometric mean titer will be reduce by 35% to a ln(geometric mean titer) of 12.18 (11.81-12.55) with SD 0.74 among those with a longer (42-day) between-dose interval. ^1, 2^ These assumed values in the <35-days dose-interval group correspond with geometric mean titers of 299,751 (95% CI 206,071-436,020), which was reported at 57 days among 15 adults aged 18-55 years given two 25mcg doses 28 days apart of the mRNA1273 that was the only published data available at the time. We also assume 90% power; that there were equal numbers of those in the <35-days interval and 42-day interval groups; and a 20% rate of loss-to-follow up. The required sample size accordingly was 300 participants.

For the second objective to detect a significant of a groups*visits interaction, we employed a simulation-based approach. The model was specified as antibody titer = (𝑏𝑏1*groups) + (*b*2*visits) + (*b*3*groups*visits interaction) + random effect. For the simulation, different values for the *b*1, *b*2 and *b*3 coefficients were used and simulation 1000 times for each combination of coefficients. A sample size of 300 had >94% power to detect an interaction coefficient (*b*3) between 0.4 and 0.8 assuming *b*1 and *b*2 both = 0.8. Despite varying the visit coefficient (*b*2) between 0.7 and 0.9, the study power remained >94%.

**REFERENCES:**

1. Barrett JR, Belij-Rammerstorfer S, Dold C, et al. Phase 1/2 trial of SARS-CoV-2 vaccine ChAdOx1 nCoV-19 with a booster dose induces multifunctional antibody responses. *Nat Med* 2021;27:279-88.
2. Anderson EJ, Rouphael NG, Widge AT, et al, Safety and immunogenicity of SARS-CoV-2 mRNA-1273 Vaccine in Older Adults. *N Eng J Med* 2020; 383:2427-38.
3. Kontopantelis E, Springate DA, Parisi R, et al. Simulation-based power calculations for mixed effects modeling: ipfpower in Stata. Journal of Statistical Software 2016;74.

**S4 APPENDIX:**

**Crude mean and SD values for antibody titers by visit and dosing intervals.**

|  | **Time interval between 1st and 2nd vaccine doses** | | |
| --- | --- | --- | --- |
|  | **<35-days** | **35-42 days** | **>42 days** |
| **IgG Spike** |  |  |  |
| 2^nd^ dose | 2.54±0.56 | 2.41±0.56 | 1.49±0.64 |
| 3 weeks | 2.95±0.08 | 2.93±0.10 | 2.88±0.07 |
| 3 months | 2.75±0.29 | 2.82±0.17 | 2.78±0.24 |
| 6-9 months | 1.84±0.63 | 1.72±0.57 | 2.21±0.44 |
| **IgG RBD** |  |  |  |
| 2^nd^ dose | 1.57±0.71 | 1.93±0.72 | 0.86±0.58 |
| 3 weeks | 3.02±0.09 | 2.95±0.22 | 2.64±0.40 |
| 3 months | 2.12±0.61 | 2.21±0.60 | 2.43±0.57 |
| 6-9 months | 0.99±0.54 | 0.71±0.48 | 0.99±0.54 |
| **IgA Spike** |  |  |  |
| 2^nd^ dose | 1.84±0.86 | 1.00±0.72 | 0.67±0.75 |
| 3 weeks | 2.27±0.70 | 2.18±0.75 | 2.22±0.70 |
| 3 months | 1.20±0.73 | 1.23±0.75 | 1.45±0.78 |
| 6-9 months | 0.65±0.56 | 0.62±0.49 | 0.74±0.60 |
| **IgA RBD** |  |  |  |
| 2^nd^ dose | 0.68±0.56 | 0.36±0.22 | 0.26±0.19 |
| 3 weeks | 1.09±0.68 | 0.78±0.61 | 0.48±0.44 |
| 3 months | 0.36±0.26 | 0.33±0.25 | 0.29±0.22 |
| 6-9 months | 0.26±0.20 | 0.23±0.14 | 0.20±0.12 |
| **IgM Spike** |  |  |  |
| 2^nd^ dose | 1.04±0.57 | 0.73±0.33 | 0.30±0.18 |
| 3 weeks | 0.94±0.64 | 0.48±0.26 | 0.55±0.50 |
| 3 months | 0.39±0.20 | 0.35±0.18 | 0.62±0.42 |
| 6-9 months | 0.33±0.15 | 0.32±0.17 | 0.28±0.13 |
| **IgM RBD** |  |  |  |
| 2^nd^ dose | 0.41±0.20 | 0.41±0.21 | 0.25±0.11 |
| 3 weeks | 0.48±0.38 | 0.29±0.13 | 0.27±0.16 |
| 3 months | 0.24±0.11 | 0.22±0.10 | 0.23±0.12 |
| 6-9 months | 0.17±0.07 | 0.17±0.09 | 0.15±0.06 |

Crude titers for serum binding antibodies to ancestral SARS-CoV-2 Spike and RBD in each group measured at the same time point post-2^nd^ dose. Data are expressed in optical density (OD) and SD.

**S5 APPENDIX.**

**Crude mean and SD values for neutralizing antibody titers by visit and dosing intervals.**

|  | **Time interval between 1st and 2nd vaccine doses** | | |
| --- | --- | --- | --- |
|  | **<35-days** | **35-42 days** | **>42 days** |
| **Ancestral** |  |  |  |
| 2^nd^ dose | 4.06±1.22 | 3.87±1.03 | 2.50±1.09 |
| 3 weeks | 5.23±0.80 | 5.27±0.75 | 6.09±0.74 |
| 3 months | 4.40±0.86 | 4.74±0.80 | 5.04±0.97 |
| 6-9 months | 2.53±0.77 | 2.52±0.89 | 3.09±0.87 |
| **B.1.351 variant** |  |  |  |
| 2^nd^ dose | 23.4±125.6 | 17.8±21.1 | 11.3±21.2 |
| 3 weeks | 142.4±136.6 | 188.2±166.2 | 208.3±267.4 |
| 3 months | 46.3±130.3 | 36.0±25.1 | 61.4±87.7 |
| 6-9 months | 16.5±18.6 | 19.9±36.7 | 22.2±25.4 |

Data are presented as crude logarithmic titers and SD needed to inhibit 50% of infection due to ancestral or Beta variants.
